# How Were Patient Safety Incidents Responded to, Investigated, and Learned From Within the English National Health Service Before the Implementation of the Patient Safety Incident Response Framework? A Rapid Review

**DOI:** 10.1097/PTS.0000000000001349

**Published:** 2025-05-09

**Authors:** Gemma Louch, Carl Macrae, Rebecca Talbot, Siobhan McHugh, Jane K. O’Hara

**Affiliations:** * School of Healthcare, University of Leeds, Leeds; † Business School, University of Nottingham, Nottingham; ‡ School of Humanities and Social Sciences, Leeds Beckett University, Leeds; § The Healthcare Improvement Studies (THIS) Institute, University of Cambridge, Cambridge, UK

**Keywords:** england, investigation, learning, patient safety, policy, reporting

## Abstract

**Objective:**

To understand how National Health Service organizations routinely responded to, investigated, and learned from patient safety incidents in England before the implementation of the Patient Safety Incident Response Framework, and to identify associated success criteria and barriers.

**Methods:**

We followed rapid review methodology and searched 2 electronic databases. We aimed to identify and synthesize literature regarding patient safety incident response, investigation, and learning within the English National Health Service, before the implementation of the Patient Safety Incident Response Framework.

**Results:**

Nineteen articles were included. A narrative synthesis generated 4 concepts: (1) a multifaceted reporting culture, (2) investigation processes, (3) the landscape of support and involvement, and (4) opportunities to learn. Barriers to incident reporting included time, task characteristics, a culture of blame, and lack of feedback. Root cause analysis was cited as the most common investigation method. Studies outlined points of support and involvement for patients and families, the importance of supporting and involving patients and families, and acknowledged contributions from patients and families may be overlooked currently. For health care staff, the need for timely and personalized support soon after an incident was emphasized. Studies underlined the limitations of current approaches to learning and improvement.

**Conclusions:**

These findings lend support to the challenges associated with health care systems’ infrastructures and strategies for responding to and learning from patient safety incidents. These challenges centre on 2 interrelated issues: the investigative challenges of rigorously conducting systems analysis and learning-oriented improvement; and the relational challenges of supporting genuine relationships of care, open and honest communication, and supportive engagement after patient safety incidents.

## BACKGROUND

With preventable patient harm affecting nearly one in 20 patients receiving medical care,^1^ improving patient safety remains a global challenge.^2^ Although there has been significant attention on patient safety over the last 20 years, limited progress in reducing the incidence of harm is recognized.^3,4^ Like health care globally, within the English National Health Service (NHS), there has been substantial focus on patient safety policy and practice. However, despite this persistent focus within the NHS in England, progress in improving patient safety has been criticized.^5^ At the same time, the landscape of patient safety policy and regulation has proliferated,^6^ leading to an expansive, complex, and fragmented web of national and local systems for reporting incidents, incident investigation, and systems of oversight and scrutiny. This makes the study of the policies guiding and practices associated with incident reporting, response, and learning, of continuing relevance to safety scholars, as well as policymakers and managers of health services.

In the English NHS, patient safety policy that directed the reporting, responding to, and learning from patient safety incidents, was first established just over 2 decades ago. In 2003, the National Patient Safety Agency established the National Reporting and Learning System.^7^ This was a voluntary scheme for the reporting of patient safety incidents, and was designed to support learning from these incidents, including sharing safety information nationally through “safety alerts,” with a view to reducing recurrence. This system established the first reporting infrastructure within the English NHS and represented the first significant national patient safety policy.

In the decade following the establishment of the National Reporting and Learning System, concerns began to emerge that incident reporting had become an “industry”; that “…reporting systems have focused on collecting and processing large quantities of incident data…”(;^8^ p.72), and that services “…collect too much and do too little.” (;^8^ p.74). Partly in response to these concerns, the Serious Incident Framework (SIF) was published and subsequently updated in 2015.^9^ This framework aimed to support health service organizations to better distinguish where to focus their resources and efforts on the most intensive investigations. This was achieved through the provision of defined thresholds, recommended investigation approaches (root cause analysis), as well as expectations for the involvement and engagement of staff, patients and families. In short, this policy aimed to reduce the “industry,” and encourage learning and action. However, criticisms relating to the “industry” of incident reporting did not subside in the wake of the SIF.^10,11^ Indeed, the need to focus more on learning than simply reporting and investigating, was highlighted again with the publication of the NHS Patient Safety Strategy in 2019.^12^ Building upon this strategy, and following a significant consultation and development exercise, the Patient Safety Incident Response Framework (PSIRF)—was published in August 2022.^13^ The PSIRF seeks to create incident response and learning processes that are more proactive, proportionate, flexible, equitable and fair.

While the topic of how health care organizations manage patient safety incidents is one that has been well studied to date, what has been less considered is what the foundations of previous policy might mean for incoming patient safety policy initiatives. Indeed, from this brief history of incident reporting policy alone, one might surmise that similar criticisms have beset all policy and practice efforts to establish and maintain reporting systems that actually support learning and improvement.

The work presented in this paper is part of a large research programme—the Response Study (https://responsestudy.leeds.ac.uk/)—that seeks to evaluate in real-time, the implementation of the PSIRF. As part of this evaluation, it was important for us to establish what the “landscape” of patient safety incident response, investigation, and learning was in health services within England, to understand how and in what ways the new policy direction effects change.

Therefore, in this rapid review, we aimed to synthesize empirical literature from the English NHS before the implementation of PSIRF, to generate evidence to further our understanding of the implementation of this significant, new patient safety policy.

Our specific objective was to:

- Understand how NHS organizations routinely responded to, investigated, and learned from patient safety incidents in England before the implementation of PSIRF and identify associated success criteria and barriers.

## METHODS

We followed the methods described by Tricco et al.^14^

### Search Methods

The review was made rapid by limiting the search to 2 electronic databases and by focusing on a narrower timeframe. We searched 2 electronic databases (MEDLINE and Embase) on July 27, 2022 on the Ovid platform. Searches were restricted to English language and studies published from 2015 onwards, in line with the most recent SIF publication.^9^

The search strategy (Appendix 1, Supplemental Digital Content 1, http://links.lww.com/JPS/A702) was informed by search terms used in previous reviews, for example, patient safety learning systems,^15^ patient safety incident reporting (^16,17^), and application of learning for patient safety.^18^ The search terms were also informed by the process for the management of patient safety incidents set out in the SIF.^9^ The search strategy was reviewed by academic researchers and the wider research programme team, which included a patient and family involvement and engagement advisor.

The search was organized into 3 blocks:

- Block 1: Terms relating to patient safety incidents (combined with OR).
- Block 2: Terms relating to incident response, investigation, and learning (combined with OR).
- Block 3: Terms relating to the English NHS (combined with OR).

Blocks 1 to 3 were combined with the AND function.

### Eligibility Criteria

Inclusion and exclusion criteria were developed and refined by the review team and are presented in Table 1. Studies from 2015 onwards about patient safety incident response, investigation, learning, and success criteria or barriers related to the NHS in England were eligible for inclusion.

**TABLE 1:** Eligibility Criteria

Inclusion Criteria	Exclusion Criteria
Reference to patient safety incidents	No reference to patient safety incidents
Content about patient safety incident response, investigation, learning, and success criteria or barriers	No content about patient safety incident response, investigation, learning, and success criteria or barriers
Related to the NHS in England	Not related to the NHS in England
Studies from 2015 onwards	Studies before 2015
Published in the English language	Not published in the English language
Published and peer-reviewed empirical studies	Not a published peer-reviewed empirical study, or full-text unavailable
Study design not restricted including qualitative, quantitative, or mixed methods studies	Review articles, opinion pieces, editorials

NHS, National Health Service.

### Study Selection

Identified articles were collated in reference management software (EndNote) and duplicates removed, then uploaded to the review software CADIMA.^19^ Study selection involved 2 levels of screening; title and abstract, and full-text. Each title and abstract were assessed according to the eligibility criteria independently by 2 reviewers (G.L., S.M., and R.T.), 2 reviewers (G.L., S.M., and R.T.) then independently assessed potentially eligible full texts. Any discrepancies regarding the eligibility of an article were resolved through discussion between the reviewing team. Reference lists of all eligible articles were checked, and additional searches were carried out through the web engine Google Scholar.

### Quality Appraisal

All eligible articles were assessed for quality using the Quality Appraisal for Diverse Studies (QuADS) tool due to the anticipated methodological diversity of included studies.^20,21^ One reviewer (R.T.) assessed all articles, with a random cross-check of 10% of included articles by a second reviewer (G.L.). The included studies had an average QuADS score of 66% (range: 41%-92%). Four studies did not mention the rationale for the data collection tool used, and 5 studies did not mention the rationale for the analytic method chosen. Four studies had limited or no mention of strengths and limitations. Nine studies did not mention that research stakeholders had been considered in the research design or conduct. No articles were excluded based on quality.

### Data Charting and Analysis

We extracted information from each article including: the authors, publication year, title, aims, sample, region, data source, study design, analysis method, and findings relating to how patient safety incidents are routinely responded to (e.g., reporting), investigated (e.g., methods used, patient and family involvement), and learned from, and associated success criteria and barriers. One reviewer (R.T.) independently extracted the information from the included articles, and one reviewer checked 10% of the data extracted (G.L.).

Descriptive techniques were used to summarize and synthesize the information.^22^ Frequent discussions within the review team took place to iteratively make sense of the information and facilitate interpretation. This narrative synthesis process developed a description of key concepts in relation to the review objectives (responding, investigating, and learning), which the findings are organized around.

## RESULTS

### Search Outcome

**FIGURE 1:**
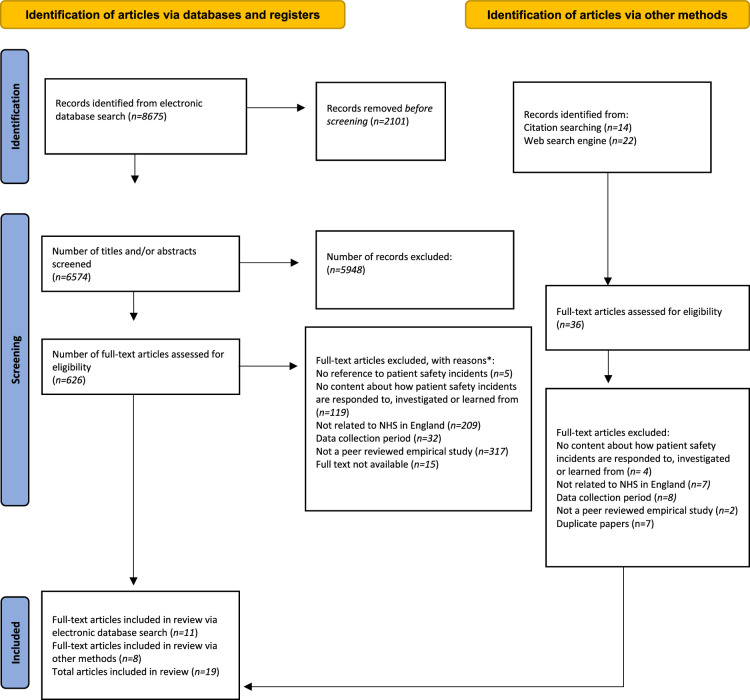
PRISMA flow diagram. PRISMA, Preferred Reporting Items for Systematic Reviews and Meta-Analyses. *Note: Articles may have been excluded for more than one reason.

The searches in MEDLINE and Embase yielded 8675 results. 2101 duplicate articles were removed before screening. Screening of titles and abstracts excluded 5948 records, and 626 articles were screened at the full-text stage. This resulted in the inclusion of 11 articles from the electronic database searches. A further 8 articles were included following citation and web engine searching, resulting in a final sample of 19 articles. Figure 1 presents details of the search and identification process.

### Summary Characteristics

A summary of article details, including study design, sample, data source, and findings, can be found in Table 2. Studies published after July 27, 2022 (electronic database search) represent eligible studies identified through web engine searching. With respect to study design, we included: 5 interview studies, 6 studies using incident databases and/or reports, 3 focus group studies, 3 questionnaire studies, 1 Delphi study, and 1 case study. The studies included varied in size and scope and spanned a range of health care services, including mental health, community, acute, maternity, and ambulance.

**TABLE 2:** Summary of Included Articles

Author	Sample	Data Source/type of Data	Study Design	Setting	Aim	Key Findings Relating to the Review Objectives
Archer and Colhoun (2018)^10^	(n=581) Clinicians. 372 consultants, 63 speciality trainees, 40 core trainees, 58 foundation trainees, 42 staff grade/speciality doctors, and 6 had other (e.g., managerial) roles	Questionnaire	Cross-sectional study	11 NHS trusts ranging from small district general hospitals to large tertiary referral centres	To assess whether clinicians recognized incidents and reported them accordingly, along with their behaviors towards reporting and their suggestions of how incident reporting might be improved	Responding, learning, and improvement: Barriers to completing incident reports: not having enough time, feeling no action would be taken, lack of feedback Organizational issues: A feeling that incident reports are completed to meet targets rather than to improve care, a feeling that completing reports may not lead to a change in practice or a solution to the problem Form structure: A feeling the form structure is inadequate, not fit for purpose or too complex A culture of blame: Many felt fear of repercussions contributed to poor reporting culture Lack of feedback: This can lead to disengagement with the process, a sense that completing incident reports does not lead to improvement
Adamson et al (2022)^23^	133 radiation incidents	DATIX data, root cause analysis meeting minutes, free text comments on patient attendance records	Retrospective mixed methods study	Multisite NHS trust	To explore trends in computed tomography radiation incidents and suggest strategies for improvement	Responding, investigating, learning, and improvement: Incidents reported on DATIX, the investigation method was usually root cause analysis. The average number of days to report an incident was 3.5 d. There was a statistically significant increase in the time taken to investigate incidents between 2015 and 2018 The investigation phase from report to investigation closure was an average of 17.9 d. Most incidents were investigated using a “system approach,” and reports highlighted the relevant action taken to try prevent reoccurrence. The root cause analysis meeting minutes highlighted themes around contributory factors, incident analysis, learning, and dissemination methods. In terms of actions, no disciplinary actions mentioned, but typically–support and further education and training for staff recommended Reporting near misses valued within the trust
Odejimi et al (2021)^24^	48 cases	Serious incident reports and process	Retrospective study	Mental Health Trust	To explore common themes emerging from root cause analysis of serious incident reports for mental health patients who died by suicide under the care of a mental health trust	Responding, investigating, learning, and improvement: Serious incident report process: Serious incident identified and reported through electronic system; decision made by head of investigations about whether the incident needs to be investigated; investigations team register incident and allocate investigator; Investigator receives terms of reference and report deadline; Investigator should read patient notes and consider questions to ask the staff involved; contact patient/next of kin to see if they would like to be involved in investigation process; arrange to interview staff involved; complete report, including recommendations and actions; send report to clinical director for approval, then on to the Investigations team; serious incidents group review the root cause analysis, if no changes required– sent on to commissioners; once approved by commissioners, case is closed; Final report disseminated to those involved, e.g.,–patients/carers, coroner. • Reports written by a senior clinician who was not involved in the care of the patient (usually within 3 mo of the event) • Investigations commence soon after the incident to avoid issues about recollection of events • Report not published until coroner’s verdict established • Root causes and other findings are disseminated in team meetings • Actions taken based on the recommendations suggested for the learning process and service changes 6 of the reports had no identified root cause
Shepard et al (2022)^25^	(n=44) participants from 3 organizational levels (i.e., executives, managers, operational staff)	Semistructured interviews	Qualitative study	3 ambulance service NHS trusts	To explore staff perceptions of patient safety in the NHS ambulance services	Responding: Reporting: Participants perceived the reporting culture as having been poor in NHS ambulance services historically, where front-line staff were fearful of reporting patient safety incidents due to the existence of a pervasive blame culture. This was perceived as contributing to an expectation and fear of punitive measures following reporting incidents. Participants indicated that these issues had since improved, staff largely feel supported by organizations to report, and a culture of blame has reduced, with a robust reporting system now in place
Archer et al (2020)^26^	(n=52) members of staff	Focus groups	Qualitative study	One NHS trust. Large specialist provider of mental and community health care	To investigate if barriers and facilitators affecting incident reporting in mental health care are consistent with factors identified in other health care settings	Responding, learning, and improvement: Frustration at the lack of learning and improvement resulting from incident reporting. Participants felt that the aims and objectives of incident reporting are sometimes unclear. Many believed that the main role of incident reports was to establish how mistakes had been made to apportion blame Participants acknowledged that local learning reports are useful and suggested there were more opportunities for this information to feed into Trust-wide improvements Time was reported as the biggest barrier to reporting incidents. Most participants believed that incident reports should be completed immediately due to the level of detail that is needed, and that this needed to be prioritized within workloads. However, it was acknowledged that immediate reporting may not always be appropriate or achievable, e.g., after a serious incident involving harm to those involved, as the immediate focus was on patient and staff wellbeing Participants expressed frustration that when completing incident reports, there was a need to replicable information that was present in other care management systems Participants described fear around the impact of incident reporting for themselves, the department and the patients in their care. The severity of the incident and individual reporting of the event were also said to impact reporting behaviors. Difficulties the trust experienced in working effectively with the Police and the Crown Prosecution Service reduced motivation to report incidents
Bovis et al (2018)^27^	(n=267) 31 consultants, 90 middle-grade doctors, 82 nurses, 33 foundation doctors, 31 allied health care professionals	Questionnaire	Cross-sectional study	5 NHS hospitals, including major trauma centre, an elective orthopedic unit, and 3 district general hospitals	To evaluate the experiences of different health care staff with adverse incident reporting and to identify factors that influence reporting of adverse incidents	Responding, learning, and improvement: 33% of staff had never reported an adverse incident. 36% of staff could recall receiving training in reporting and 23% of staff reported confidence knowing how the reporting process works. Of those who had completed a report, 28% of staff reported receiving feedback and 33% of staff felt the primary issue had been resolved, and 60% of staff noticed repeated similar incidences Training and feedback identified as important factors to improve confidence in and use of reporting
Serou et al (2021)^28^	(n=45) medical and nonmedical operating room staff	Semi-structured interviews	Qualitative study	Large NHS trust composed of 5 teaching hospitals providing multispecialty surgical procedures, including emergency and major trauma	To explore what support operating room staff actually received after surgical incidents and what other kinds of support would have helped them in moving forward	Responding, investigating, learning, and improvement: 3 key themes including (1) sources of support - peers, friends, and family, (2) the timing of the support, (3) challenges of the investigation process Importance of personalized support soon after the incident, and for senior clinicians to be proactive in offering support in line with a culture of openness and support. Some staff felt reluctant to seek support – “Sign of weakness” Investigation processes - themes of isolation and frustration evident The need for collaborative approaches to promote cross-disciplinary learning after incidents emphasized
Berry et al (2021)^29^	73 endoscopy patient safety incidents	Patient safety incident information and outcomes related to endoscopy	Prospective study	Endoscopy department of a large NHS trust	To describe a 3-tiered approach to investigation to facilitate appropriate action, shared learning, and timely disclosure to patients as mandated in the UK health system by the DoC	Responding, investigating, learning, and improvement: Hospital-level root cause analysis performed in 6 cases, mini root cause analysis in 12 cases, structured judgment report in 2 cases, and for 53 cases, examined by the endoscopy lead. Findings presented in an endoscopy user group meeting. Introduction of the 3-tiered approach facilitated investigation, action and learning, and timely communication with patients and relatives
Canham et al (2018)^30^	(n=21) health care stakeholders	Workshops	Case study	Not applicable	To compare the processes and outputs of a current practice root cause analysis-based incident analysis and a STAMP analysis on the same medication error incident	Investigating: Original investigation undertaken through root cause analysis. Teams usually carry out investigations alongside clinical caseloads People involved: 2 patient safety coordinators and a team of 7 clinical staff, chaired by an assistant chief nurse Data used: interviews and documentation analyzed using timeline, fishbone diagram, and incident decision tree tools Limitation of root cause analysis: not modelling the relationships between system components and not building a model of the system
Martin et al (2021)^31^	(n=70) health care staff, (n=18) patients/family members	Narrative interviews	Qualitative study	6 NHS organizations. 3 acute hospital Trusts, 2 community and mental health care Trusts, and one ambulance Trust	To offer new insights into organizations’ responses to concerns and complaints, in relation to patient and staff satisfaction and organizational failure	Responding and investigating: Incident investigations were principally concerned with establishing why the incident had happened or, in the case of near misses, what nearly happened, focusing on contributory factors and root causes aiming to reduce the likelihood of reoccurrence. Participants sometimes found that investigations did not do justice to their concerns but generally understood the rationale for incident investigations. In addition participants felt that systems currently in place were often not fit for purpose to deal with the concerns raised. Incident investigations having features such as terms of reference and strict timelines for reporting built into the design, ensured investigations focused on the aims of identifying underlying causes and reducing risk
Robbins et al (2021)^32^	22 learning team investigations; 22 root cause analysis investigations	Patient safety incident records	Retrospective mixed methods evaluation	Large University NHS trust	To explore the potential opportunities and challenges to disseminating and implementing learning team approaches versus root cause analysis	Investigating, learning, and improvement: Incidents investigated using traditional root cause analysis generated an average of 3.5 actions, and 30% of actions were graded as system-focused actions In root cause analysis discussions a significant amount of time concentrated on establishing exactly “what went wrong”. Discussions focused on the specific incident/event and considered the “why” to a lesser extent The language used to describe root cause analysis included concepts around “blame” and “mistake”
Wood et al (2023)^33^	(n=29) mental health Trusts	Questionnaire	Cross-sectional study	29 mental health trusts	To examine the current patient safety practices in a sample of mental health trusts	Responding and investigating: Reporting: 28 of the 29 trusts used a commercial database to report patient safety events. 14 trusts solely used DATIX; 13 solely used Ulysses. One trust used both. 28 trusts provided training in how to report patient safety events, the trust that did not provide training used the Ulysses database 27 out of 29 trusts reported that they used the root cause analysis method principally, and the majority of Trusts provided training on the method 16 trusts provided protected time to investigators, and 21 had processes in place to oversee the competency of investigators
Bakhbakhi et al (2017)^34^	(n=11) bereaved parents who had experienced a perinatal death	Focus group	Qualitative study	Not applicable	To investigate bereaved parents’ views on involvement in the perinatal mortality review process	Responding, learning, and improvement: Most participants were unaware that a formal perinatal mortality review process took place after the death of a baby. The need for transparency was highlighted. Parents wanted to know the perinatal mortality review was taking place and to have the lessons learned clearly communicated. Parents felt that parental input to the review process should be optional and flexible and that the review process should capture both the clinical and emotional aspects. Parents suggested the review process should include positive aspects of care
Bakhbakhi et al (2018)^35^	(n=27) health care professionals	Focus groups	Qualitative study	Maternity hospitals at 2 NHS trusts	To explore whether health care professionals would accept or support parent engagement in the perinatal mortality review process	Responding, learning, and improvement: Health care professionals indicated that not all parents know formal meetings take place and agreed that parents’ involvement in the perinatal mortality review process is useful and necessary Themes included: parental engagement; formal follow-up; critical structure of perinatal mortality review meeting; coordination and streamlining of care; advocacy for parents, including role of the bereavement care lead; and requirement for training and support for staff to enable parental engagement
Bakhbakhi et al (2019)^36^	(n=25) stakeholder panel of clinical and academic experts in perinatal loss and neonatal and bereavement care	2-round Delphi technique Round 1 included a national consensus workshop, and round 2 online questionnaire	Consensus study	Not applicable	To develop core principles and recommendations for parental engagement in perinatal mortality review in the UK	Responding, learning, and improvement: 96% agreed that a face-to-face explanation of the perinatal mortality review process was of critical importance; 72% indicated that parents should be offered the opportunity to nominate a suitable advocate; 92% believed that responses to parents’ comments should be formally documented; 96% suggested that it was vital for action plans to be translated into lessons learned and that this process should be monitored, and 100% of stakeholders voted that a plain-English summary should be produced for parents
Berry et al (2022)^37^	33 endoscopy patient safety incidents	Review of cases of significant harm related to endoscopy notified DATIX	Retrospective study	Endoscopy department of a large NHS trust	To describe the experience of DoC following patient safety incidents related to endoscopy and to offer recommendations on improving compliance across other areas of clinical practice	Responding and investigating: Verbal apology documented in 23 cases (70%), written notification offered or sent in 20 cases (61%) Verbal apologies were reported as timely (median 0.5 d), there were delays in sending out letters to patients and families (median 33 d) Evidence of inviting patients or families to present questions for the investigation in all 20 written notifications, in 2 cases questions or specific concerns were presented, and there was documented evidence in 7 cases that the patient or family were sent the outcome of the investigation, and in one case had been invited to a meeting for further discussion
Brummell et al (2021)^38^	(n=222) NHS Secondary Care Trusts	Quality account data and reports	Qualitative and quantitative	NHS Secondary Care Trusts	To review how NHS trusts are using the LfDs framework to learn and prevent potentially preventable deaths	Responding, investigating, learning, and improvement: 98 out of 222 NHS Secondary Care Trusts reported all 6 statutory elements of the LfDs reporting framework. The number of case record reviews or investigations undertaken relative to the number of patient deaths in individual trusts ranged from 0.2%-100% of deaths (average 43.7%). 111 out of 222 trusts indicated the use of structured judgment reviews alone or in combination with other forms of investigation or review. Trusts not using structured judgment reviews used other methods such as: confidential enquiry into stillbirths and deaths in infancy framework, root cause analysis, and preventable incidents survival and mortality methodology 105 out of 222 trusts referred to assessment of impact, many trusts used audits and/or quality improvement projects to understand whether actions are implemented 37 out of 222 trusts referred to the involvement of families/carers either in the investigation process or in shared learning or that they communicated with/support/engage/consider families/carers after patient had died 106 out of 222 trusts had shared or planned to share the learning more widely within their own organization, 17 out of 222 trusts had shared or planned to share the learning outside their organization
Lalani et al (2023)^39^	(n=40) managers and clinicians	Semistructured interviews	Qualitative study	5 NHS organizations. 3 acute trusts. 2 community/mental health trusts	To examine how contextual factors influence implementation of the LfDs policy and the ability of the programme to achieve its goals	Responding, investigating learning, and improvement: There was variation in the mortality review process across the Trusts. Typically, a senior clinician screened all deaths. When quality of care concerns were raised at the screening stage the records were then sent for further in-depth reviews by senior clinicians. Cases were often reviewed in-depth if the death was unexpected or if the death was in line with the LfDs selection criteria. In-depth reviews used methods such as structured judgment review. Learning from reviews was typically shared at the directorate and/or organization level. Many participants suggested that the policy had resulted in improved communication and engagement with bereaved relatives. Joint mortality and morbidity meetings viewed as an important forum to facilitate learning across different departments and clinical teams. Some Trusts had channels to communicate key messages to front-line staff, including learning seminars, workshops, and written materials
Olagundoye et al (2022)^40^	(n=10) maternity staff members; (n=2) consultants, (n=3) specialist registrars, (n=5) midwives	Semistructured interviews	Qualitative study	Tertiary university maternity teaching hospital	To explore maternity staff’s experiences with the incident reporting and investigation process, with specific reference to its impact on trust management leadership and the organizational process	Responding and investigating: 4 key themes including (1) the human response to adverse outcomes, characterized by guilt, self-blame, and anxiety, (2) lack of trust in local risk management processes, derived from poor communication, (3) limited leadership visibility, (4) lack of structured support, leaving staff relying solely on colleagues for support Emotion-focused response to patient safety incidents (e.g., guilt, self-blame, anxiety). The majority of participants reported not receiving feedback or updates on the investigation into the adverse incident that they were involved in

DoC, Duty of Candour; LfDs, Learning from Deaths; NHS, National Health Service; STAMP, Systems Theoretic Accident Modelling and Processes.

## NARRATIVE SYNTHESIS

We present our synthesis under 4 concepts: (1) a multifaceted reporting culture, (2) investigation processes, (3) the landscape of support and involvement, and (4) opportunities to learn.

## A MULTIFACETED REPORTING CULTURE

Following an incident being identified, the decision to report is a central response “activity,” and incidents are reported through electronic incident reporting systems.^10,23–25^ Studies identified challenges, opportunities, and recommendations pertaining to reporting behaviors and implications, which are outlined below.

### Time and Task Characteristics

“Time” constraints coupled with existing workload priorities and “specific task characteristics” such as the structure of forms not being fit for purpose were highlighted as problematic factors contributing to underreporting.^10,26^ Regarding time, in a qualitative study focused on mental health care settings, when reflecting on the capacity to complete incident reports, participants suggested incident reporting should be prioritized within workloads to enable an appropriate in-depth report.^26^ In the same study, although immediate reporting was preferred by most participants, in some instances, it was acknowledged that immediate completion of incident reports may not always be appropriate or achievable, as immediate focus is on the wellbeing of patients and staff.^26^

Findings from a cross-sectional study of health care staff across multiple hospitals identified training and feedback as important factors to improve confidence in and use of reporting, as those who received training were more likely to submit reports.^27^

### A Culture of Blame

A fear of adverse consequences and the importance of health care staff feeling psychologically comfortable to report was a recurrent theme across health care services.^10,25,26^ From the ambulance service perspective, a qualitative study across 3 ambulance service NHS trusts, including executives, managers, and operational staff, referred to a poor reporting culture historically within NHS ambulance services in part due to the existence of a pervasive blame culture and fear of punitive measures for reporting incidents. Over time, these issues were said to have improved towards a culture where staff feel supported by their organizations to report incidents, with robust reporting systems now in place.^25^

In a qualitative study focused on mental health care settings participants felt that the aims and objectives of incident reporting were sometimes unclear, many participants involved in the focus groups believed the main role of incident reports was to establish how mistakes had been made to apportion blame.^26^ A culture of blame was also identified as a theme in a cross-sectional survey of clinicians across 11 hospitals regarding incident reporting behaviors, further reinforcing its persistent existence across health care services.^10^

### Discourses Around Feedback

Lack of feedback was identified across numerous studies as a significant barrier to reporting incidents that may result in missed opportunities for learning by limiting people’s motivation to report.^10,26,27^ A cross-sectional study evaluated the experience of incident reporting and factors influencing reporting among health care staff.^27^ Of those staff who had previously completed an incident report, only 28% had received feedback on the action taken as a result of the report; 33% felt the primary issue had been resolved; and crucially, 60% recognized repeated similar subsequent incidents.^27^ Indeed, the quality of feedback received was deemed important for meaningful future engagement with the incident reporting process in a cross-sectional survey of clinicians.^10^

## INVESTIGATION PROCESSES

Following the reporting stage and a decision that the incident requires further investigation,^24,32^ root cause analysis was cited as the most common method for conducting investigations across studies and health care services, involving set timelines and processes that typically focus on identifying underlying causes and reducing risk.^23,24,29–33^

A questionnaire study describing current investigatory capability within mental health trusts reported that 27 out of 29 trusts responding to the questionnaire specified root cause analysis as their primary method of investigating serious incidents.^33^ This study also outlined challenges with the current practice of investigating serious incidents and recommendations, for example, training and education investigators received being below the recommended standard and the need to provide protected time and subject matter supervision to staff undertaking investigations.^33^

Regarding the length of time for an investigation, a retrospective mixed methods study of computed tomography–related radiation incidents within a multisite trust, reported a statistically significant increase in the time taken to investigate incidents between 2015 and 2018, and posited the longer investigation timeframe may be indicative of a more comprehensive inquiry being undertaken.^23^ The same study reported that root cause analysis meeting notes included information about contributory factors, incident analysis and learning and dissemination method, and typically, no disciplinary actions were referenced, but staff support and education/training recommended.^23^

Studies comparing root cause analysis with approaches to incident investigation less utilized in health care currently reinforced the limitations of the root cause analysis approach. For instance, a study comparing a learning team investigation approach to the traditional root cause analysis investigation approach described that in root cause analysis discussions, a significant amount of time concentrated on establishing exactly “what went wrong” focused on the specific incident/event, and considered the “why” to a lesser extent.^32^ Moreover, a study comparing the processes and outputs of root cause analysis and a systemic accident analysis approach—Systems Theoretic Accident Modelling and Processes analysis identified the root cause analysis not modelling the relationships between system components and not building a model of the system as a key limitation.^30^

## THE LANDSCAPE OF SUPPORT AND INVOLVEMENT

### Patients and Families

Several studies outlined the points of support and involvement for patients and families, the importance of supporting and involving patients and families, and acknowledged contributions from patients and families may be overlooked currently.^24,34–38^

Two studies focused on the learning from deaths (LfDs) programme. In a documentary analysis of LfDs reports (2017/2018), only 17% of Trusts referred to involvement of bereaved families and carers in the investigation process or in shared learning, or that they communicate with, support, engage, or consider bereaved families and carers after a patient has died.^38^ A qualitative study including managers and clinicians across 5 NHS Trusts (3 acute trusts and 2 community/mental health trusts) recognized that further work is required to involve families in incident investigation^39^ but that broadly, the LfD policy had facilitated improved communication and engagement with bereaved relatives, often through medical examiners.

A prospective study of endoscopy patient safety incidents described the introduction of a 3-tiered approach to investigation in a large teaching hospital.^29^ The study suggested its introduction facilitated opportunities to ensure timely communication with patients and relatives.^29^ A related retrospective study of endoscopy patient safety incidents and the experience of Duty of Candour concluded that the Duty of Candour process is still a challenge for clinicians and patient safety teams.^37^ A verbal apology was documented for 70% of cases, written notification was offered/sent in 61% of cases, and delays in sending out letters to patients and families were reported. There was evidence of inviting patients or families to present questions for the investigation in all written notifications, but typically, this invitation did not translate into more substantial involvement.

Several connected studies addressed maternity services and parental engagement in the perinatal mortality review process.^34–36^ In a focus group study of bereaved parents, many participants were unaware a formal perinatal mortality review process takes place after the death of a baby. Most parents supported the potential for parental involvement, the need for an individualized approach was emphasized and for parental input to be optional and flexible.^34^ Transparency deemed was essential, and parents felt the clinical and emotional aspects of care should be captured and lessons learnt should be clearly communicated.^34^

In a focus group study, health care professionals acknowledged it was evident not all parents know formal meetings take place.^35^ Participants recognized parental engagement as a priority and the potential for the parental perspective to produce clinically useful information not documented in medical notes, and the importance of a flexible, personalized approach to involvement that was sensitive to timing.^35^ It was acknowledged that some parents would need short-term and long-term support, and the need for terms of reference for parental engagement was suggested. Health care professionals discussed potential challenges to engagement, including how to approach questions that were unanswerable or unexpected, requests for further investigations, parental relationship issues, and involving vulnerable parents. Looking ahead and considering recommendations, the study highlighted that support from hospital trusts and central support from the government is needed to address challenges and enable parental engagement to be implemented in a meaningful and practical way, and also emphasized the need for staff to be trained and supported to facilitate parental engagement.

A subsequent Delphi study generated 12 core principles for parental engagement that outlined the importance of face-to-face explanations, opportunity for parents to nominate an advocate, responses to parents’ comments being formally documented, action plans and lessons learned to be monitored, and the need for plain-English summaries.^36^

### Health Care Staff

The need for timely and personalized support soon after an incident was emphasized.^28,40^ In a qualitative study involving medical and nonmedical operating room staff at one large NHS trust, participants described the importance of support from colleagues and peers and indicated that debriefs with team members were helpful.^28^ Participants described variations in the support staff received. In comparison with surgeons and anesthetists, nurses and operating department practitioner staff, seemed to receive limited or no support. Surgeons and anesthetists perceived openly discussing incidents in mortality and morbidity meetings as useful, but there was recognition that some members of the multidisciplinary team were not present at these meetings, and some surgeons and anesthetists expressed concerns that seeking support may be viewed as a sign of weakness. Pertaining to investigation processes, themes of isolation and frustration were evident.^28^ Indeed, feelings of isolation during investigation processes were also evidenced in a qualitative study exploring maternity staff’s experiences with incident reporting and investigation processes.^40^ Staff described a lack of updates and feedback on investigations into incidents they were involved in, and lack of structured support for staff was an important theme. Support was described as being either absent, or when it was provided, not sufficient or timely, and in some cases inappropriate. This study recommended that staff receive timely feedback and updates and structured support, and for risk management leaders to be visible throughout reporting and investigation processes.

## OPPORTUNITIES TO LEARN

The potential for and importance of incident reports and investigations driving improvement in practice was evidenced in studies across health care settings^10,26,29^ as well as the value of reporting near misses,^23^ but crucially, studies emphasized significant limitations of current approaches to learning and improvement.^10,26,38,40^ For example, completing incident reports not leading to improvement was highlighted in a cross-sectional survey of clinicians across 11 hospitals regarding incident reporting behaviors under the theme “lack of feedback.”^10^

Studies predominantly described “local” approaches within organizations to sharing and disseminating learning, for example: multidisciplinary committees and regular clinical meetings,^29^ team debriefs,^26^ meetings/events, case studies on intranet, safety alerts, newsletters,^38^ joint mortality and morbidity meetings, seminars, workshops and materials to communicate messages to front-line staff.^39^

In terms of opportunities for the future, from the maternity perspective, when considering parental engagement in perinatal mortality reviews and facilitating learning, health care professionals suggested there may be information that could be learned from reviews that is not currently captured/documented and shared with parents and staff.^35^

## DISCUSSION

Analyzing, responding to, investigating, and learning from patient safety incidents has become established as one of the foundational strategies for safety improvement within health care.^2,41,42^ Considerable policy attention, organizational resource and staff time are committed to incident response activities.^6,43^ These activities remain some of the most important in determining how patients, families, and health care staff are supported and engaged with following a safety incident, as well as how effectively health care systems improve in the aftermath of failure.

This review has explored relevant literature that has engaged with these issues in the context of the English NHS, when these activities of incident response and learning were determined by a particular, and now historical policy framework—the SIF.^9^ The findings of this review illustrate the ways in which the SIF attempted to create a standardized set of conditions and processes for organizational analysis, investigation, response, and learning—but, in practice, also created a set of constraints in the ways that organizations implemented incident response and did not adequately address a range of complications and challenges that are inherent to the processes of learning from incidents. Broadly, these centre on 2 interrelated issues: the investigative challenges of rigorously conducting systems analysis and learning-oriented improvement; and the relational challenges of supporting genuine relationships of care, open and honest communication, and supportive engagement after incidents.

To effectively learn from safety incidents, organizations need to understand what those incidents mean for the reliability and safety of the systems and practices through which care is delivered. The literature indicates that this has been a challenge in many instances. Pressures to meet reporting deadlines can reduce the time available for in-depth investigation along with a limited methodological repertoire focused primarily on root cause analysis methods.^23,29–33^ Furthermore, constraints in available training and expertise of those investigating incidents can limit the explanatory depth of incident analysis.^33^

Limited evidence of systems analysis and process modelling reinforces a general concern that incident analysis and investigation have historically struggled to engage with underlying systemic issues. Without rigorously analyzing the systems from which incidents emerge, incident response is necessarily limited to considering safety issues one event at a time. Moreover, without effectively engaging with the underlying systems and the processes and practices that deliver care, efforts to learn from incidents can be limited to exhortation and communication of findings, through localized meetings or other reports, rather than the substantive and collaborative reforms to care practices that can sometimes be required to prevent events recurring.

Alongside these challenges in learning from incidents, the literature indicates wider challenges of supportively engaging with patients, families, and health care staff—after safety incidents. These concerns have been more widely identified as a key issue in patient safety,^44–48^ and this review identifies some particular issues in relation to incident investigation and response. Principle among these is a simple lack of engagement in many instances, with incident response either not engaging with patients and families or resorting to highly formalized and ritualized processes.^24,34–38^ Likewise, similar challenges were evidenced in feeding back, informing and supporting health care staff after events, accentuated by a potential professional reluctance of health care staff to seek support.^28,40^

## CONCLUSIONS

These findings lend support to the emerging consensus that health care systems’ infrastructures and strategies for responding to and learning from safety incidents have considerable gaps.^8,49,50^ Health care faces unique challenges in learning from incidents, both in terms of the large scale of harmful events that occur across health care, and the hugely impactful personal, emotional and relational nature of many of these events and outcomes. These experiences illustrate the importance of redoubling efforts to focus on building more effective systems, policies, and practices to support health care’s infrastructure of learning. Incidents provide a key route into safety improvement and already represent a foundational piece of our safety architecture. This now needs to more effectively engage with the practices and strategies that support rigorous systems analysis and improvement, and genuine care and compassion to all those involved in safety incidents. This review highlights that considerably greater research attention is warranted to the interrelated issues identified, if we are to make more rapid progress in this important area.

## Supplementary Material

SUPPLEMENTARY MATERIAL
