# Supplementary material for: How Were Patient Safety Incidents Responded to, Investigated, and Learned From Within the English National Health Service Before the Implementation of the Patient Safety Incident Response Framework? A Rapid Review
Source: J Patient Saf. 2025 May 9;21(5):e42–55. doi: 10.1097/PTS.0000000000001349 (PMC12266792; doi:10.1097/PTS.0000000000001349)
Supplement: SUPPLEMENTARY MATERIAL [file pts-21-e42-s001.docx]

**Appendix 1**

**Medline search strategy**

| **Block 1: Patient safety incidents** |
| --- |
| Patient adj safety adj incident* |
| Harm* |
| Serious adj incident* |
| Serious adj2 incident* |
| Severe adj incident* |
| Severe adj2 incident* |
| Death |
| Unexpected adj incident* |
| Unexpected adj2 incident* |
| Notifiable |
| Unexplained |
| Adverse adj event* |
| Adverse adj2 event* |
| Near adj miss* |
| Critical adj incident* |
| Critical adj2 incident* |
| Clinical adj incident* |
| Clinical adj2 incident* |
| Never adj event* |
| *Medical Errors/ |
| *Patient Safety/ |
| Error* |
| Negligen* |
| Sentinel adj event* |
| Sentinel adj2 event* |
| Incident* |
| Accident* |
| Iatrogenic |
| ((adverse or avoidable or preventable or unsafe or safet*) adj2 (event* or outcome* or complication* or death* or effect* or reaction* or accident* or injur*)) |
| ((medica* or diagnostic or therapeutic or administrat* or dispens* or prescri*) adj2 (error* or mistake* or fault*)) |
| (patient* adj2 (risk* or incident* or accident* or harm*)) |
| **Block 2: Patient safety incident management, responses, investigation and learning** |
| *Risk Management/ |
| ((Incident or safety or error) adj (report* or respon* or investigat* or manag* or system*)) |
| ((Incident or safety or error) adj2 (report* or respon* or investigat* or manag* or system*)) |
| Report* adj system* |
| NRLS |
| LFPSE |
| National adj reporting adj2 learning adj system* |
| Learn adj2 patient adj safety adj events adj service* |
| Feedback |
| Learning adj system* |
| Safety adj monitor* |
| Safety adj polic* |
| Response adj polic* |
| Investigat* adj polic* |
| **Block 3: English NHS** |
| *”Delivery of Health Care”/ |
| Health service* |
| NHS |
| English adj NHS |
| English adj health adj service* |
| Health adj service* adj2 England |
| NHS adj2 England |
| Healthcare |
